# Regulation of Cullin-RING ubiquitin ligase 1 by Spliceosome-associated protein 130 (SAP130)

**DOI:** 10.1242/bio.20134374

**Published:** 2013-06-26

**Authors:** Lucia Cordero-Espinoza, Thilo Hagen

**Affiliations:** Department of Biochemistry, Yong Loo Lin School of Medicine, National University of Singapore, Singapore 117597, Singapore

**Keywords:** Cullin-RING ligase 1, SAP130, p27

## Abstract

Cullin-RING ubiquitin ligases (CRLs) mediate the ubiquitination of numerous protein substrates and target them for proteasomal degradation. The function of CRLs is under tight regulation by Cullin-binding proteins. It has been reported that the Spliceosome-associated protein 130 (SAP130/SF3b-3) binds to several Cullin proteins, yet it remains unknown whether SAP130 plays any role in regulating the function of CRLs. Here, we report that SAP130 overexpression reduces the binding of adaptor protein Skp1 and substrate receptor Skp2 to Cul1, whereas it has no effect on CAND1 binding to Cul1. Overexpression of SAP130 decreases the degradation rate of p27, a protein substrate of the SCF^Skp2^ ligase. Interestingly, silencing of SAP130 also inhibits the degradation of p27, suggesting a dual role for SAP130 in the regulation of SCF activity. We hypothesized that the regulatory role of SAP130 could extend to other CRLs; however, overexpression of SAP130 is unable to affect the protein stability of the Cul2 and Cul3 substrates, HIF-1 and NRF-2. SAP130 binds to Cul1, Cul2 and Cul4 with similar affinity, and it binds to Cul3 more strongly. SAP130 localizes in both the nucleus and the cytoplasm. Hence, the inability of SAP130 to regulate Cul2 and Cul3 CRLs cannot be explained by low binding affinity of SAP130 to these cullins or by subcellular sequestration of SAP130. We propose a novel role for SAP130 in the regulation of SCF, whereby SAP130 physically competes with the adaptor protein/F-box protein for Cul1 binding and interferes with the assembly of a functional SCF ligase.

## Introduction

Protein degradation is critical for the regulation and maintenance of cellular functions such as cell signaling, transcriptional regulation and cell cycle progression (Petroski and Deshaies, 2005). The majority of proteins in the cell are degraded via the ubiquitin-mediated proteolytic pathway (Ciechanover et al., 1984). Ubiquitin is a conserved 76 amino acid polypeptide that is covalently attached to protein substrates as a poly-ubiquitin chain to target them for degradation (Pickart and Fushman, 2004). The ubiquitination reaction is catalyzed by three classes of enzymes: a ubiquitin-activating enzyme (E1), a ubiquitin-conjugating enzyme (E2) and a ubiquitin ligase (E3). The E3 ligase plays a pivotal role as it facilitates the transfer of ubiquitin onto a determined protein substrate, thus providing the substrate specificity of the reaction (Pickart and Cohen, 2004).

The largest family of E3 ligases in eukaryotes are the Cullin-RING ligases (CRLs) (Petroski and Deshaies, 2005). Each CRL is nucleated by a member of the cullin family, which in human cells consists of seven members (Cul1, 2, 3, 4A, 4B, 5 and 7). The substrate recognition module of the CRL assembles at the N-terminus of the cullin protein (Wu et al., 2000). It encompasses a substrate receptor and, with the exception of Cul3-based CRLs, an adaptor protein that links the substrate receptor to the complex (Petroski and Deshaies, 2005).

SCF (Skp1-Cullin1-F-box) complexes are the most well characterized E3 ligases (Willems et al., 2004). They consist of Cul1, Skp1, and an F-box domain-containing protein that serves as the substrate receptor (Deshaies, 1999). Cul2-based ligases on the other hand recruit VHL-box proteins as substrate receptors via the Elongin C/B adaptor (Kamura et al., 2004); while Cul3-based ligases bind to BTB domain-containing proteins, which serve a dual role as substrate receptors and adaptors (Pintard et al., 2004).

Despite their diverse structure, CRLs are regulated by conserved mechanisms that involve cullin-associated proteins. In a process termed neddylation, UBC12 conjugates the ubiquitin-like protein Nedd8 to cullins. This covalent modification is required for the *in vivo* function of Cul1, Cul2 and Cul3 (Bosu and Kipreos, 2008). Cullin neddylation is reversed by the COP9 signalosome (CSN) in a reaction known as de-neddylation (Lyapina et al., 2001). CRLs are also regulated by CAND1 (cullin-associated and neddylation dissociated), a highly conserved protein that prevents adaptor and Nedd8 binding to Cullin and therefore inhibits CRLs (Zheng et al., 2002; Liu et al., 2002; Goldenberg et al., 2004). Both CSN and CAND1, although being negative regulators of CRLs *in vitro*, are required for efficient CRL-dependent ubiquitination *in vivo* (Schwechheimer et al., 2001; Doronkin et al., 2003; Feng et al., 2004; Chuang et al., 2004).

Spliceosome associated protein 130 (SAP130) is a component of the SF3b RNA splicing complex as well as STAGA and TFTC transcription complexes (Das et al., 1999; Golas et al., 2003; Brand et al., 2001; Martinez et al., 2001). Interestingly, SAP130 has sequence homology with DDB1, the Cul4 protein adaptor (Li et al., 2006). In a recent study, SAP130 was reported to associate with Cul1, Cul2 and Cul4A ligases (Menon et al., 2008). Notwithstanding, unlike CSN and CAND1, the role of SAP130 as a putative regulator of CRL function has not been thoroughly investigated. Our study suggests that SAP130 plays a novel regulatory role in the assembly and function of CRLs that appears to be specific to Cul1-based complexes.

## Results

### SAP130 competes with Skp1-F-box protein for binding to Cul1

**Fig. 1.**
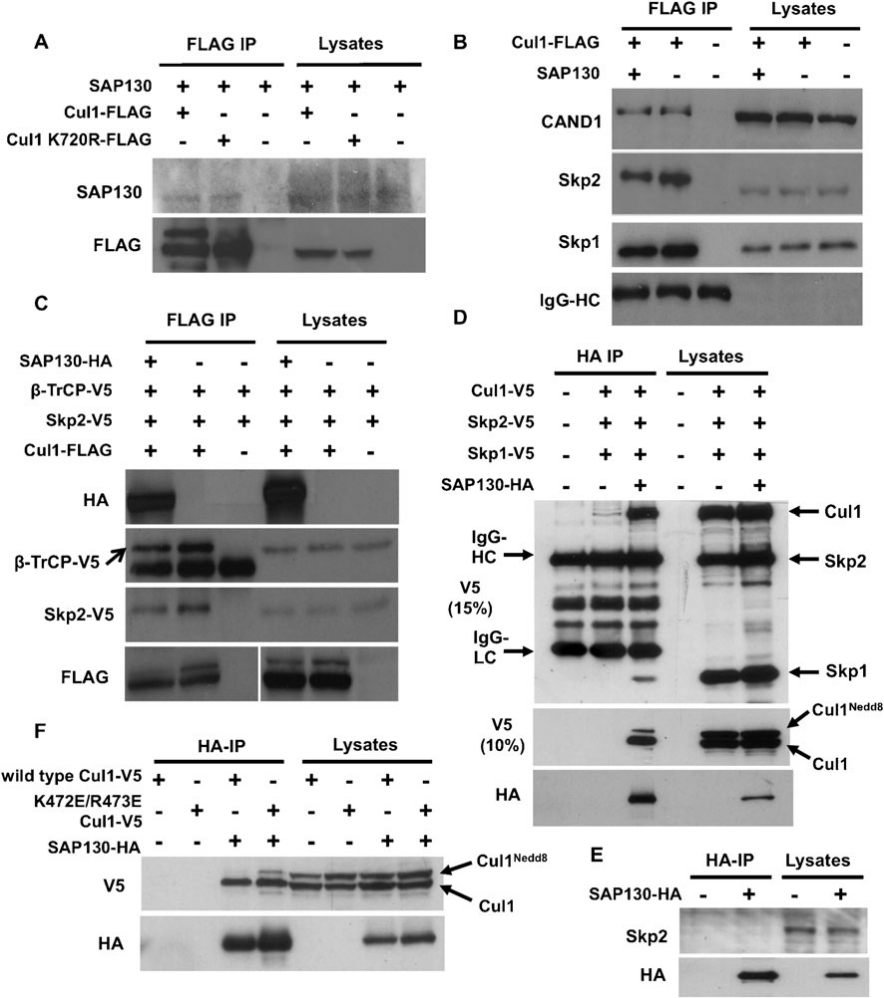
SAP130 competes with Skp1-F-box protein for Cul1 binding. (**A**) HEK293T cells were co-transfected with SAP130 and either Cul1-FLAG wild-type or Cul1 K720R-FLAG mutant. Lysates were FLAG immunoprecipitated and analyzed for SAP130 binding. (**B**) HEK293T cells were co-transfected with Cul1-FLAG and either SAP130 or empty vector. Lysates were FLAG immunoprecipitated and blotted with the indicated antibodies. (**C**) HEK293T cells were transfected as indicated with Skp2-V5, β-TrCP-V5, Cul1-FLAG and SAP130-HA. Following FLAG immunoprecipitation, binding of Skp2-V5 and β-TrCP to Cul1 was checked. (**D**) HEK293T cells were co-transfected with V5-tagged plasmids of Skp1, Skp2 and Cul1, and either SAP130-HA or empty vector; HA-immunoprecipitation was carried out. The immunoprecipitates and lysates were run on a 15% SDS gel to visualize Cul1-V5, Skp2-V5 and Skp1-V5 on the same Western blot by V5 immunoblotting. To visualize both the neddylated and unneddylated forms of Cul1-V5, the samples were re-run on a 10% SDS gel (middle panel). (**E**) HEK293T cells were transfected with SAP130-HA or empty vector, followed by immunoprecipitation of cell lysates using HA-agarose beads. Binding of endogenous Skp2 to SAP130-HA was detected by Western blotting of immunoprecipitates and lysates with Skp2 antibody. (**F**) HEK293T cells were transfected with the indicated expression plasmids. Cell lysates were subjected to HA immunoprecipitation and were blotted with V5 and HA antibodies.

We first sought to corroborate the previous work by Menon et al. regarding the binding of SAP130 to CRL complexes (Menon et al., 2008). To this end, we made use of the archetypal cullin ligase, Cul1, to test for interaction with SAP130. We co-overexpressed SAP130 with either wild-type Cul1-FLAG or a mutant form of Cullin1, Cul1-FLAG K720R, in which the neddylation site is mutated and which cannot therefore be neddylated. Following FLAG immunoprecipitation, SAP130 was found to bind to both wild-type and neddylation-deficient Cul1 (Fig. 1A). Accordingly, SAP130 was confirmed to physically interact with a member of the CRLs, and neddylation of the cullin ligase appears to be non-essential for binding of SAP130.

Treatment of cells with the small molecule inhibitor MLN4924, which effectively inactivates Cullin-RING E3 ligases by blocking neddylation, had no effect on the protein levels of SAP130 (data not shown). This suggests that SAP130 is not a substrate of CRLs. As a cullin-binding protein, SAP130 could instead represent a regulator of CRL structure and activity. We hypothesized that SAP130 may be able to regulate the assembly of CRL components, namely substrate receptor, adaptor protein and other regulatory proteins such as CAND1. To investigate this, Cul1-FLAG was immunoprecipitated in HEK293T cells in the presence of co-transfected SAP130 or empty vector and relative levels of co-immunoprecipitating CRL components were assessed (Fig. 1B). Skp2 is one of the most abundant and best-characterized F-box proteins. We thus blotted for co-immunoprecipitating Skp2 to assess the effect of SAP130 on substrate receptor association with Cul1. Interestingly, we consistently observed decreased binding of Skp2 in the presence of overexpressed SAP130 (Fig. 1B). In agreement with this, we also noticed a slight reduction in binding of the Cul1 adaptor protein Skp1, which is known to recruit Skp2 to the SCF complex. These results suggest that association of SAP130 with Cul1 could displace the Skp1-Skp2 complex from the SCF ligase. On the other hand, the binding of CAND1, a protein that regulates SCF complex assembly, did not seem to be affected by SAP130 overexpression (Fig. 1B). CAND1 has been reported to prevent the binding of Skp1 and Skp2 to Cul1 (Zheng et al., 2002). Consequently, the mechanism through which SAP130 reduces binding of Skp2 and Skp1 to Cul1 cannot be mediated by increased recruitment of CAND1.

To confirm the effect of SAP130 on the binding of substrate receptor to Cul1, we investigated another well-characterized Cul1 substrate receptor, β-TrCP. A similar co-immunoprecipitation experiment as the one shown in Fig. 1B was carried out. Cells were co-transfected with Skp2-V5 and β-TrCP-V5, with the objective of comparing binding affinities of these substrate receptors to Cul1 upon SAP130 overexpression. As expected, overexpression of SAP130 reduced Skp2-V5 binding to Cul1, corroborating the result with endogenous Skp2 in Fig. 1B. Importantly, SAP130 reduced binding of β-TrCP-V5 to Cul1 in a similar degree as it reduced binding of Skp2-V5 (Fig. 1C), suggesting that SAP130 inhibits the interaction between substrate receptors and the Cul1 ligase.

Our results suggest that SAP130 competes with Skp1-Skp2 for Cul1 binding. Competition between SAP130 and Skp1-Skp2 entails mutually exclusive binding of these proteins to Cul1 and lack of association between the competing proteins. However, it has previously been reported that SAP130 co-immunoprecipitates with Skp2 and Skp1 (Menon et al., 2008). Because of this contradiction, we deemed it necessary to compare the binding affinity of SAP130 to Cul1, Skp2 and Skp1. In order to do this, we first constructed a SAP130 expression plasmid with a C-terminal Haemogglutinin (HA) tag that would allow us to immunoprecipitate SAP130. HEK293T cells were then co-transfected with Cul1-V5, Skp2-V5 and Skp1-V5 and either SAP130-HA or an empty vector. Cul1, Skp2 and Skp1 all contained V5 epitope tags to rule out any bias in antibody sensitivity during Western blotting and validly compare the relative binding affinities of these proteins to SAP130. Interestingly, whereas Cul1-V5 was found to co-immunoprecipitate with SAP130-HA, only very weak binding of Skp1-V5 to SAP130-HA was observed, despite similar expression levels of Cul1-V5 and Skp1-V5 (Fig. 1D). In the experiment shown, we were unable to evaluate Skp2-V5 binding to SAP130-HA because Skp2-V5 co-migrated with heavy chain IgG in the immunoprecipitate samples. We therefore measured binding of endogenous Skp2 to SAP130-HA by immunoprecipitation (using a rabbit Skp2 antibody that does not react with mouse IgG). As shown in Fig. 1E, no binding of endogenous Skp2 to SAP130 was observed. Taken together, these results are in agreement with the hypothesis that SAP130 competes with Skp2 and Skp1 for Cul1 binding.

Our results in Fig. 1D indicate that neddylation of the Cul1 protein does not prevent its interaction with SAP130, as we observed binding of both neddylated (slower migrating) and unneddylated (faster migrating) Cul1-V5 to SAP130-HA (Fig. 1D). However, the ratio of neddylated to unneddylated Cul1-V5 in the SAP130-HA immunoprecipitates was somewhat lower compared to the cell lysate (compare lane 3 and 6 in Fig. 1D). This could be explained by partial deneddylation of Cul1 during the immunoprecipitation reaction. To confirm binding of both neddylated and unneddylated Cul1 to SAP130 we utilized the K472E/R473E Cul1 mutant. This mutant exhibits higher basal neddylation levels as a consequence of reduced binding to CSN and thus reduced deneddylation (Chua et al., 2011). Following SAP130 immunoprecipitation, we observed more binding of K472E/R473E Cul1^Nedd8^ than the wild-type Cul1^Nedd8^ (Fig. 1F). Taking together the results from Fig. 1A,D,F, we conclude that neddylation does not regulate the interaction between Cul1 and SAP130.

### SAP130 regulates the degradation of the SCF^Skp2^ substrate, p27

Given the observed inhibitory effect of SAP130 on Skp1-F-box protein binding to Cul1, we sought to investigate whether the presence of SAP130 translates into altered activity of the SCF complex. We assessed the protein stability of p27, a well-known substrate of Cul1 that is recognized by Skp2 (Carrano et al., 1999). Protein stability of p27 acts as a good read-out of SCF activity because it reflects the ability of the ligase to target its substrates for proteasomal degradation.

**Fig. 2.**
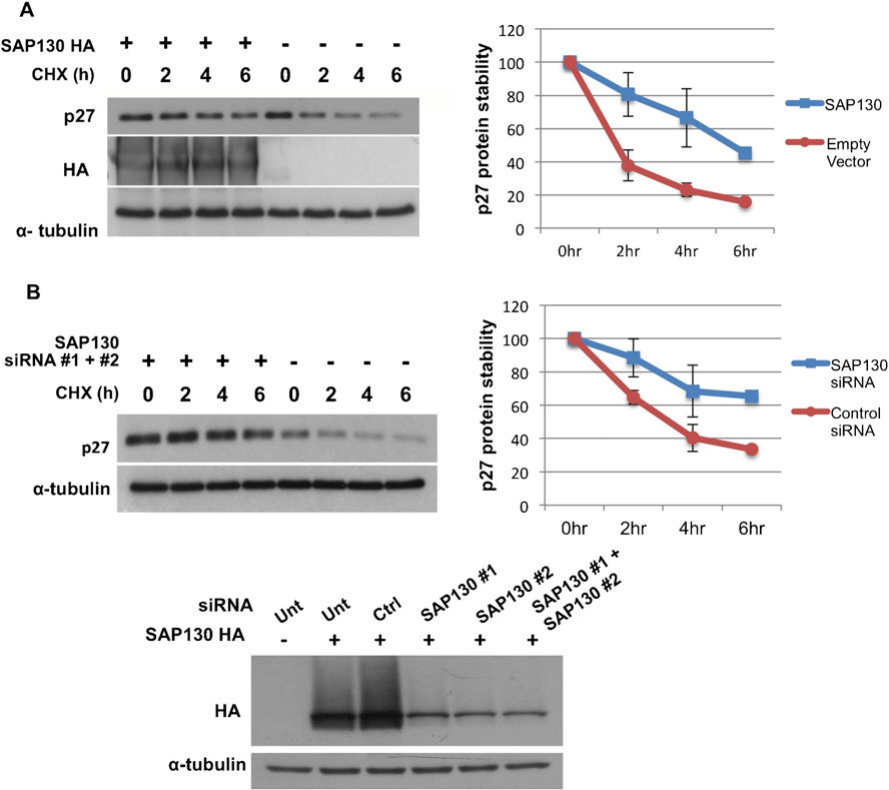
SAP130 regulates p27 degradation. (**A**) SAP130-HA or empty vector-transfected HEK293T cells were treated with 40 µM cycloheximide (CHX) and lysed at time 0 h, 2 h, 4 h and 6 h. Lysates were blotted with p27 antibody. Three independent experiments were analyzed for protein abundance with the densitometry tool of the software ImageJ. Results were expressed as p27 protein values/tubulin protein values and as percent of time 0 h. Error bars denote s.e.m, *n* = 3. (**B**) HEK293T cells were transfected with two dsiRNA oligos targeting SAP130 or with a negative control dsiRNA oligo. Three days post transfection cells were treated with 40 µM CHX and lysed at time 0 h, 2 h, 4 h, 6 h. Lysates were blotted with p27 antibody. Densitometry results were expressed as p27 protein values/tubulin protein values and as percent of time 0 h. Error bars denote s.e.m, *n* = 2. Efficiency of SAP130 knockdown by the two dsiRNA oligos was determined by co-transfecting cells with SAP130-HA plasmid and either SAP130 oligo #1, SAP130 oligo #2 or SAP130 oligo #1 + SAP130 oligo #2.

Cells were transfected with either SAP130 or empty vector and were treated with cycloheximide (CHX) to inhibit mRNA translation. Cells were lysed at different time intervals and examined for abundance of endogenous p27 protein. Notably, overexpression of SAP130 reduced the rate of p27 degradation (Fig. 2A, left panel). Based on densitometry measurements of three independent experiments, SAP130 transfection increased the half-life of p27 by more than twofold (Fig. 2A, right panel). This result suggests that SAP130 competition with Skp2-Skp1 for Cul1 binding is physiologically significant, as impeded SCF assembly translates into delayed degradation of the p27 substrate.

In order to confirm the role of SAP130 in p27 degradation, SAP130 was knocked down by siRNA and p27 stability was assessed (the knockdown efficiency of the siRNA oligos was confirmed by silencing of transfected SAP130 in HEK293T cells) (Fig. 2B, bottom panel). It is predicted that a knockdown experiment should provide results that contrast with an overexpression experiment. Yet, surprisingly, the rate of degradation of p27 upon SAP130 knockdown was reduced, similarly to when SAP130 was overexpressed (Fig. 2B, left panel and right panel). This result raises the possibility of a dual role of SAP130 in regulating SCF ligases (see Discussion).

### SAP130 does not regulate the function of Cul2 and Cul3-based CRLs despite binding affinity and co-localization

We speculated that the effect of SAP130 on SCF activity could be a conserved regulatory mechanism for other Cullin-RING ligases. To test this, we assessed the effect of SAP130 overexpression on the protein stability of HIF-1α and NRF2, two established substrates for Cul2- and Cul3-dependent ubiquitination, respectively (Maxwell et al., 1999; Kobayashi et al., 2004).

**Fig. 3.**
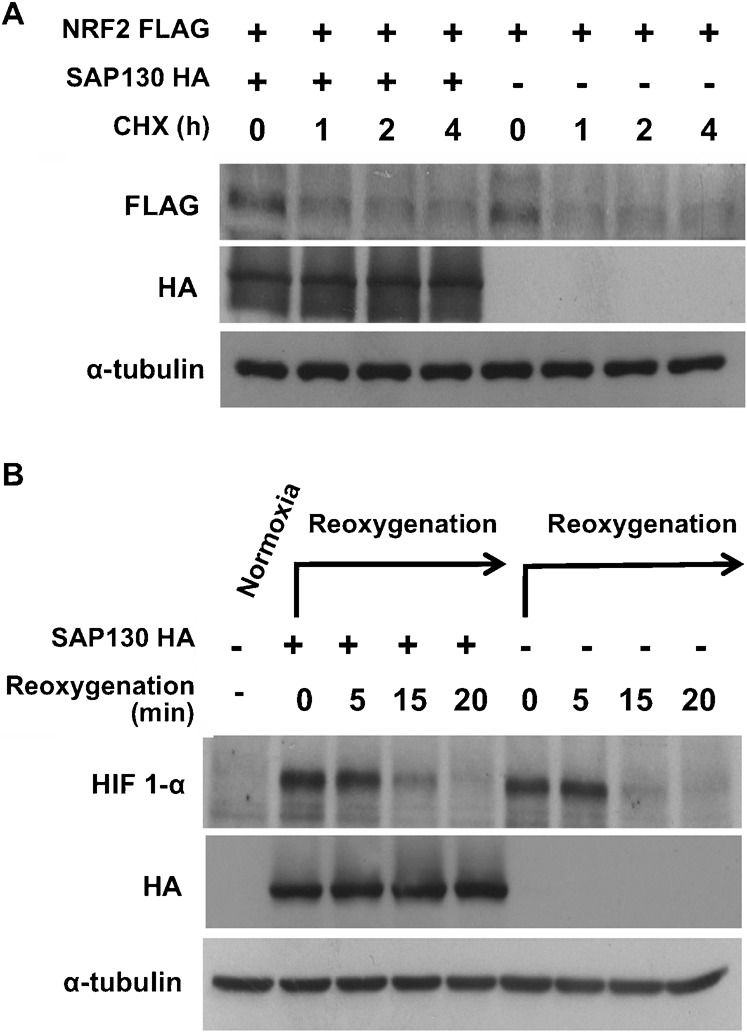
SAP130 does not regulate the function of Cul2 and Cul3-based CRLs. (**A**) NRF-2 was co-transfected with SAP130 or empty vector. Cells were treated with 40 µM CHX and lysed at time 0 h, 2 h, 4 h, 6 h. Lysates were analyzed for NRF-2 degradation rate. (**B**) SAP130-HA-transfected cells were incubated at 1% oxygen using a Pro-ox oxygen controller and Pro-ox *in vitro* chamber (BioSpherix, Redfield, NY) for 4 hours to stabilize HIF-1α. Cells were lysed before incubation as a normoxia control and upon re-oxygenation at 0 min, 5 min, 15 min and 20 min.

The NRF2 stability experiment was first performed on endogenous NRF2 but visualization of protein bands proved to be difficult due to low sensitivity of the NRF2 antibody. A FLAG-tagged plasmid of NRF2 was thus transfected into cells along with either SAP130 or empty vector and protein stability was monitored over time. Surprisingly, in contrast to the p27 result, the degradation rate of NRF2-FLAG was unaffected by SAP130 (Fig. 3A).

The HIF-1α protein was stabilized by incubating cells in a hypoxic chamber and the rate of HIF-1α degradation was measured at different time points upon re-oxygenation. Similarly to NRF2, the degradation of HIF-1α was not affected by SAP130 overexpression (Fig. 3B).

**Fig. 4.**
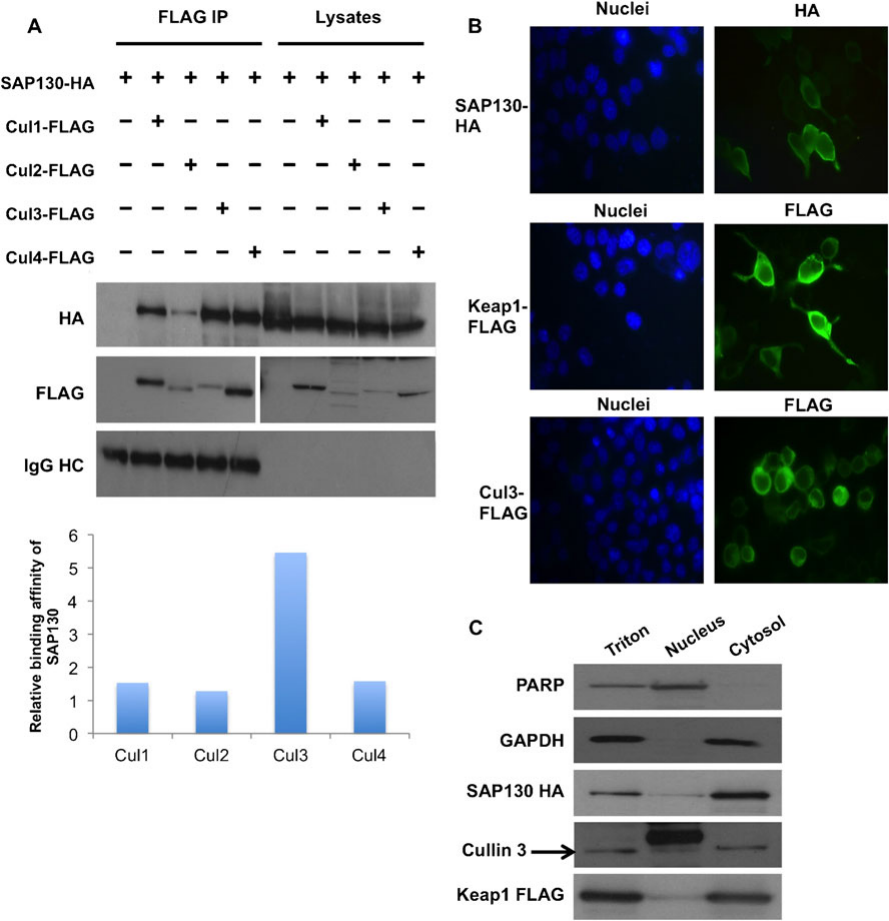
Cullin binding affinity and sub-cellular localization of SAP130. (**A**) HEK293T cells were co-transfected with SAP130-HA and FLAG plasmids of Cul1, Cul2, Cul3 or Cul4. Binding affinity of SAP130 to each Cullin was analyzed following FLAG immunoprecipitation. Protein abundance was measured by densitometry with ImageJ software. Cullin-bound SAP130 protein was normalized to the amount of immunoprecipitated Cullin. Results were plotted in a bar graph. (**B**) SAP130-HA, Keap1-FLAG or Cul3-FLAG were transfected into HEK293T cells and analyzed for *in vivo* sub-cellular localization via immunocytochemistry. Representative microscopy pictures were taken. (**C**) HEK293T cells were transfected with SAP130 and Keap1-FLAG and were subjected to cell fractionation as described under Materials and Methods. Sub-cellular localization of SAP130-HA, Keap1-FLAG and endogenous Cul3 was determined by Western blotting. PARP was used as a nuclear marker and GAPDH as a cytoplasmic marker.

We sought to investigate further the apparent lack of effect of SAP130 on the function of non-SCF CRLs. SAP130 has been reported to bind to Cul1, Cul2 and Cul4; and is speculated to bind to Cul3 as well (Menon et al., 2008). We hypothesized that SAP130 binds with differing affinities to each ligase, resulting in varied degrees of CRL regulation. Based on our results, SAP130 would be expected to bind with high affinity to Cul1 but not to Cul2 and Cul3. We transfected cells with SAP130-HA and either FLAG-tagged Cul1, Cul2, Cul3, Cul4 or empty vector. FLAG-immunoprecipitation was carried out and the levels of co-immunoprecipitating SAP130 were assessed (Fig. 4A). The expression levels of the transfected Cullins varied considerably. We therefore quantified the amount of Cullin-associated SAP130 protein and normalized it by the amount of immunoprecipitated Cullin. Interestingly, we found that SAP130 binds with similar affinity to Cul1, Cul4 and Cul2; while it binds more strongly to Cul3 (Fig. 4A).

These results were surprising because, contrary to our previous data, they predicted a regulatory effect of SAP130 on all the CRLs, particularly the Cul3 ligase. As a possible explanation, we thought that the regulatory role of SAP130 could be limited by subcellular localization. SAP130 has been reported to localize to the nucleus (Gocke et al., 2005), while the ubiquitination of NRF2 by the Keap1-Cul3 ligase is known to occur in the cytoplasm (Itoh et al., 2003). Immunocytochemistry of cells transfected with SAP130-HA, Cul3-FLAG and Keap1-FLAG showed that, as expected, Cul3 and in particular Keap1 localized predominantly to the cytoplasm. However, quite unexpectedly, SAP130 was found in both the nucleus and the cytoplasm (Fig. 4B). To confirm this, we performed a cell fractionation experiment wherein SAP130-HA and Keap1-FLAG were transfected into the cells and fractions for the nucleus and cytosol were isolated. Western blotting for nuclear PARP and cytosolic GAPDH indicated that the fractions were pure (Fig. 4C). Interestingly, SAP130 was restricted to the cytoplasm, with only a small percentage found in the nucleus (Fig. 4C). Cul3 and Keap-1, in accordance with the immunocytochemistry results, were predominantly localized to the cytoplasm. It thus appears that SAP130 can co-localize with the Cul3-Keap1 ligase.

In conclusion, the moderate-to-high affinity of SAP130 to Cul2 and Cul3, as well as the localization of SAP130 in both nuclear and cytoplasmic compartments cannot account for the lack of effect of SAP130 on the degradation of Cul2 and Cul3 substrates. This suggests that SAP130 indeed does not regulate Cul2 and Cul3 CRLs, although the reason for the lack of effect of SAP130 is currently unknown.

## Discussion

Cullin-associated proteins sustain the activity of Cullin-RING ubiquitin ligases (CRLs). The SAP130 protein was reported to interact with various CRL complexes, suggesting a plausible role in the regulation of CRLs. In this study, we provide evidence that SAP130 functions as a regulator of Skp1-Cullin1-F-box protein (SCF) activity. We found that SAP130 binds to Cul1 and that overexpression of SAP130 inhibits binding of the adaptor and substrate receptor module Skp1-Skp2 to Cul1. Furthermore, this translates into a decreased degradation rate of the SCF^Skp2^ substrate p27. The inhibitory effect of SAP130 also extends to binding of another substrate receptor, β-TrCP, to Cul1. This suggests that SAP130 may play a general role in the regulation of SCF assembly. This is of significance as SCF ligases mediate the degradation of proteins involved in regulation of cell cycle, activation of transcription, signal transduction and DNA replication.

SAP130 is part of the U2 small nucleotide ribonucleoprotein (snRNP) associated complex SF3b involved in pre-mRNA slicing (Das et al., 1999; Golas et al., 2003). However, the specific role of SAP130 is currently not completely understood. Interestingly, SAP130 has significant sequence homology with the Cul4 adaptor protein DDB1 (Li et al., 2006). Recently, Menon et al. showed that SAP130 co-purifies with CSN1, one of the eight subunits of the CSN complex (Menon et al., 2008). In their studies, the authors showed that SAP130 binds to Cul1, Cul2 and Cul4A to form tertiary complexes with CRLs. SAP130 was reported to bind to both the N-terminus and C-terminus of cullins (Menon et al., 2008).

In our study we confirmed the ability of SAP130 to interact with Cul1. Neddylation of Cul1 is neither necessary nor obstructive for SAP130 binding. We found that SAP130 binds to both the neddylation-deficient K720R Cul1 mutant and the neddylation-enhanced K472E/R473E Cul1 mutant. This is in clear contrast with the interaction between CAND1 and Cul1, as CAND1 binds to unneddylated Cul1 and neddylation dissociates CAND1 from Cul1 (Liu et al., 2002).

We hypothesized that, as a cullin-binding protein, SAP130 might play a regulatory role in the CRL pathway. We found that binding of SAP130 to Cul1 results in decreased binding of Cul1 adaptor Skp1 and substrate receptors Skp2 and β-TrCP. The impaired assembly of the SCF^Skp2^ complex affected the activity of the ligase, as the degradation rate of its substrate p27 decreased. Based on these observations, we suggest a model of mutually exclusive binding of SAP130 and adaptor/substrate receptor complex to cullin. This model is in apparent disagreement with the reported ability of SAP130 to from tertiary complexes with CRLs and to co-precipitate endogenous Skp1 and Skp2 (Menon et al., 2008). However, using an approach unbiased to antibody sensitivity, we compared binding affinities and found that SAP130 interacts strongly with Cul1 whereas the interaction with Skp1 and Skp2 is significantly weaker or undetectable, respectively.

It has been proposed that cycles of CRL assembly and disassembly are required to sustain the activity of CRLs (Cope and Deshaies, 2003). Cullin-associated proteins CAND1 and CSN act as negative regulators of CRL *in vitro*; yet, they appear to be essential for the function of the ligase *in vivo*. The *in vivo* requirement of CAND1 and CSN is attributed to the regulation of CRL assembly and substrate receptor exchange (Pierce et al., 2013; Zemla et al., 2013; Wu et al., 2013). Our results showed that both knockdown and overexpression of SAP130 caused a delay in p27 degradation. It is therefore possible that akin to CAND1, endogenous levels of SAP130 promote the exchange of different substrate receptors in the cell. Knockdown of SAP130 had no significant effect on the interaction between Skp1/Skp2 and Cul1 measured by immunoprecipitation (data not shown). However, absence of SAP130 would be expected to affect the dynamic exchange, and not necessarily the steady state levels, of Cul1-bound F-box proteins. Our results suggest that SAP130 regulates Cul1 E3 ligase activity in a complex manner and SAP130 does not simply function by competing with Skp1/F-box proteins for binding to Cul1.

Based on our findings, the regulatory role of SAP130 appears to be restricted to Cul1-based CRLs. SAP130 did not affect the degradation rate of HIF-1α and NRF-2, substrates of Cul2 and Cul3, respectively. This was despite interaction of SAP130 with both Cul2 and Cul3 as well as localization of SAP130 in both the nucleus and cytoplasm. Ubiquitination of NRF2 by the Keap1-Cul3 ligase occurs in the cytoplasm (Itoh et al., 2003), while HIF-1α is ubiquitinated in both cytoplasm and nucleus in a cell-type specific manner (Berra et al., 2001). It therefore remains unclear why SAP130 is unable to exert any regulatory effect on Cul2 and Cul3 CRLs.

In conclusion, SAP130 is a Cullin-interacting protein that binds to Cul1, Cul2, Cul3 and Cul4, and localizes in both the nucleus and cytoplasm. SAP130 appears to only regulate the activity of Skp1-Cullin1-F-box E3 ligases; it competes with Skp1-F-box proteins for binding to Cul1 and negatively affects the ability of the ligase to mediate substrate degradation. This is evidenced by the increase in p27 protein stability upon SAP130 overexpression. Interestingly, lack of SAP130 also stabilizes p27. SAP130 may be involved in regulatory cycles of CRL assembly that sustain the activity of the ligase.

## Materials and Methods

### Cell culture and transfection

HEK293T cells were grown at 37°C and 5% CO_2_ in Dulbecco's modified Eagle's medium (Invitrogen), supplemented with 10% fetal bovine serum (HyClone), l-glutamine (Invitrogen) and penicillin/streptomycin (Invitrogen). DNA plasmids were transiently transfected in sub-confluent HEK293T cells with GeneJuice® Transfection Reagent (Novagen) according to the manufacturer's protocol.

### Plasmid constructs

The SAP130-HA plasmid used in this study was generated by PCR amplifying a HindIII/SacII SAP130 DNA fragment from SAP130-pCMVSPORT6 plasmid and cloning it into a HA-pcDNA3 plasmid. The SAP130 fragment was then excised as HindIII/XbaI to include the HA tag at the C-terminus and cloned back into the SAP130-pCMVSPORT6.

### Immunoblotting

Cell were washed with ice-cold PBS and lysed with Triton X-100-containing lysis buffer (see Culbert et al. for composition (Culbert et al., 2001)). Lysates were pre-cleared by centrifugation and equal amounts of protein were loaded in SDS-PAGE gels for protein separation. The proteins on the gel were transferred overnight onto nitrocellulose membranes for Western Blot analysis. The following antibodies were used to probe the membranes: goat anti-SF3B3 (Santa Cruz Biotechnology), goat polyclonal anti-Skp2 (Santa Cruz Biotechnology), rabbit polyclonal anti-Skp 1 (Cell Signaling), goat polyclonal anti-CAND1 (Santa Cruz Biotechnology), mouse monoclonal anti-p27 (BD Biosciences), mouse monoclonal anti HIF-1α (BD Biosciences), rabbit polyclonal anti-NRF2 (Santa Cruz Biotechnology), mouse monoclonal anti-α-tubulin (Molecular Probes), mouse monoclonal anti-V5 (Serotec), mouse monoclonal anti-FLAG (Sigma), rat monoclonal anti-HA (clone 3F10; Roche).

### Immunoprecipitation

Anti-FLAG M2 agarose beads (Sigma) or protein G-sepharose beads (Amersham Biosciences) coupled to anti-HA monoclonal antibody were used for immunoprecipitation. Lysates from transfected cells grown in 60 mm, 100 mm or 15 cm plates were pre-cleared by centrifugation and added to the beads. Samples were tumbled at 4°C for 2 hours and washed four times with NP40 lysis buffer containing 20 mM Tris, pH 7.5, 50 mM NaCl, 0.5 mM EDTA, 5% glycerol, 0.5% NP40, and once with buffer containing 50 mM Tris, pH 7.5.

### siRNA-mediated gene silencing

For siRNA transfections, RNAi Max Lipofectamine (Invitrogen) was used as transfection agent according to the manufacturer's instructions. The following pre-designed siRNA duplexes (IDT) at final concentration of 20 nM were used: SAP130: HSC.RNAI.N012426.12.2_2nmS (SAP130 siRNA #1) and HSC.RNAI.N012426.12.2_2nmA (SAP130 siRNA #2). Cells were lysed three days after siRNA transfection.

### Immunofluorescence

Cells were grown on cover slips that were pre-treated with poly-D-lysine. Two days after transfection, the cells were fixed with 4% paraformaldehyde and then permeabilized using 0.1% Triton X-100. After blocking with 5% serum and incubation with primary and secondary antibodies, the cover slips were mounted on slides using Vectashield with DAPI (Vector Laboratories) and inspected by fluorescence microscopy.

### Cell fractionation

Cells were lysed with hypotonic buffer (10 mM Tris, pH 7.5, 10 mM KCl, 1 mM EDTA, 1 mM EGTA, 0.1% β-mercaptoethanol and protease inhibitor cocktail (Roche)) and subjected to two consecutive freeze/thaw cycles. Following centrifugation at 4000 rpm, the supernatant and pellet were separated as cytoplasmic and nuclear fractions respectively. The cytoplasmic fraction was spun further at 13200 rpm to pellet heavy membrane organelles. The nuclear fraction was washed twice by resuspension with PBS and centrifugation at 4000 rpm. Nuclear proteins were eluted with high salt buffer (containing 20 mM Tris, pH 7.5, 420 mM NaCl, 1 mM EDTA, 1 mM EGTA, 25% glycerol, 0.1% β-mercaptoethanol and protease inhibitor cocktail (Roche)) by mixing for 20 min at 4°C. After centrifugation at 13200 rpm, nuclear proteins were recovered in the supernatant.
